# Dendritic Cell Subpopulations Are Associated with Prognostic Characteristics of Breast Cancer after Neoadjuvant Chemotherapy—An Observational Study

**DOI:** 10.3390/ijms242115817

**Published:** 2023-10-31

**Authors:** Agnieszka Łazarczyk, Joanna Streb, Anna Glajcar, Anna Streb-Smoleń, Przemysław Hałubiec, Kacper Wcisło, Łukasz Laskowicz, Diana Hodorowicz-Zaniewska, Joanna Szpor

**Affiliations:** 1 Department of Pathomorphology, Jagiellonian University Medical College, 31-501 Cracow, Polandjoanna.szpor@uj.edu.pl (J.S.); 2 Department of Oncology, Jagiellonian University Medical College, 31-501 Cracow, Poland; 3 University Centre of Breast Disease, University Hospital, 31-501 Cracow, Poland; 4 Department of Pathomorphology, University Hospital, 30-688 Cracow, Poland; 5 Department of Oncology, Maria Sklodowska-Curie National Research Institute of Oncology, 31-115 Cracow, Poland; 6 Doctoral School of Medical and Health Sciences, Jagiellonian University Medical College, 31-530 Cracow, Poland; 7 Clinical Department of Gynecology and Gynecological Oncology, University Hospital, 30-688 Cracow, Poland; 8 General, Oncological and Gastrointestinal Surgery, Jagiellonian University Medical College, 31-501 Cracow, Poland; diana.hodorowicz-zaniewska@uj.edu.pl; 9 Department of General Surgery, University Hospital, 31-501 Cracow, Poland

**Keywords:** breast cancer, dendritic cells, neoadjuvant chemotherapy, CD123, CD1a, DC-LAMP, DC-SIGN

## Abstract

Breast cancer (BC) is the most prevalent malignancy in women and researchers have strived to develop optimal strategies for its diagnosis and management. Neoadjuvant chemotherapy (NAC), which reduces tumor size, risk of metastasis and patient mortality, often also allows for a de-escalation of breast and axillary surgery. Nonetheless, complete pathological response (pCR) is achieved in no more than 40% of patients who underwent NAC. Dendritic cells (DCs) are professional antigen-presenting cells present in the tumor microenvironment. The multitude of their subtypes was shown to be associated with the pathological and clinical characteristics of BC, but it was not evaluated in BC tissue after NAC. We found that highe r densities of CD123^+^ plasmacytoid DCs (pDCs) were present in tumors that did not show pCR and had a higher residual cancer burden (RCB) score and class. They were of higher stage and grade and more frequently HER2-negative. The density of CD123^+^ pCDs was an independent predictor of pCR in the studied group. DC-LAMP^+^ mature DCs (mDCs) were also related to characteristics of clinical relevance (i.e., pCR, RCB, and nuclear grade), although no clear trends were identified. We conclude that CD123^+^ pDCs are candidates for a novel biomarker of BC response to NAC.

## 1. Introduction

Breast cancer (BC) remains the most frequently diagnosed malignancy worldwide with an incidence of 55.9 per 100,000 in developed and 29.7 per 100,000 in developing countries and is the leading cause of cancer death in women [1].

There are well-known prognostic and predictive factors, such as hormone receptor (estrogen receptor (ER), progesterone receptor (PR)) and human epidermal growth factor receptor 2 (HER2) status, as well as tumor proliferative index measured by Ki-67 expression, which influence the management and clinical outcome in BC. Standard clinicopathological prognostic factors include patient age, disease stage, tumor grade, tumor type, margin status and lympho-vascular status [2].

Neoadjuvant therapy refers to systemic treatment of BC prior to definitive surgical therapy. The purpose of administering it is to downstage the extent of the disease in the breast and regional lymph nodes and to provide information on the response to direct adjuvant therapies. Downstaging may allow less extensive surgery of the breast and axilla, thus avoiding the risks associated with breast reconstruction in patients able to undergo breast-conserving surgery in place of mastectomy, improving cosmetic outcomes, and reducing postoperative complications, such as lymphoedema [3,4,5,6,7,8].

However, it should be noted that hormone receptor positive, HER2-negative cancers are less likely to respond to neoadjuvant chemotherapy (NAC) than other biological subtypes [9,10,11,12,13].

Regarding NAC regimens, the most frequently used contain anthracyclines and/or taxanes, although cyclophosphamide/methotrexate/5-fluorouracil (CMF) may be used in selected patients [14].

HER2-positive tumors can be treated with trastuzumab and pertuzumab, paired with taxane chemotherapy and anthracycline- or platinum-based chemotherapy, while triple-negative BC (TNBC) is treated with dose-dense anthracycline and taxane-based chemotherapy [15].

The residual cancer burden (RCB) calculator provides a standardized approach to assess the extent of residual invasive disease in the tumor bed and axillary lymph nodes after neoadjuvant therapy. Scores calculated using this tool were shown to be predictive of relapse-free survival at 10 years, and when broken down into four classes (RCB-0, which is essentially synonymous with pathological complete response (pCR), RCB-I, RCB-II and RCB-III) can be used to stratify the risk of recurrence by the extent of residual disease and may help to guide the selection of subsequent systemic therapy [16,17].

Dendritic cells (DCs) are a type of professional antigen presenting cell (APC), able to induce T-cell mediated immunological response against pathogens, thus initiating adaptive immunity. They are considered to be the most potent APCs, possessing a large number of pattern recognition receptors (PRRs) on their surface [18,19].

DCs are of hematopoietic origin and their differentiation is regulated by cytokine Flt3L (FMS-like tyrosine kinase 3 ligand). Once differentiated, they migrate to sites of infection and pathological tissues, where they seek foreign antigens. Upon contact with antigens, DCs mature and migrate to lymphoid organs, where they present antigens in association with major histocompatibility complex (MHC) molecules in specific CD4^+^ and CD8^+^ T cells [20,21,22].

There are different DC subtypes, namely conventional DCs (cDCs), plasmacytoid DCs (pDCs) and monocyte-derived DCs [23].

In general, an increased number of tumor-infiltrating DCs was linked to diminished recurrence rates and improved survival rates in cancer patients [24,25,26,27]. However, in BC patients, higher infiltration of pDCs was correlated with a poorer prognosis [28,29].

In studies that found a correlation between the number of DCs in the tumor area and an improved clinical outcome, the importance of DC maturation has been shown [30,31].

The activation status of DCs is based on the expression of different superficial antigens. Dendritic-cell-specific intercellular-adhesion-molecule-3-grabbing non-integrin (DC-SIGN, CD209) and dendritic-cell-lysosome-associated membrane glycoprotein (DC-LAMP, CD208) are nonspecific indicators of mature DCs, whereas CD123 is the marker of immature pDCs and CD1a is expressed on both immature and mature DCs [32,33,34,35].

Our previous studies indicate that density and local composition of various subtypes of DCs (classified by different superficial antigens) is associated with certain morphological and molecular features in a primary and metastatic BC setting, which renders them as prognostic factors, both favorable and unfavorable. Furthermore, the local preponderance alone of distinct DCs subtypes was associated with certain tumor behavior and lower or higher burden of sentinel lymph nodes (SLNs) [36,37,38].

The primary objective of this study was to evaluate the relationship between the expression of superficial DC antigens and the substantial prognostic factors of BC after NAC.

## 2. Results

**Figure 1:**
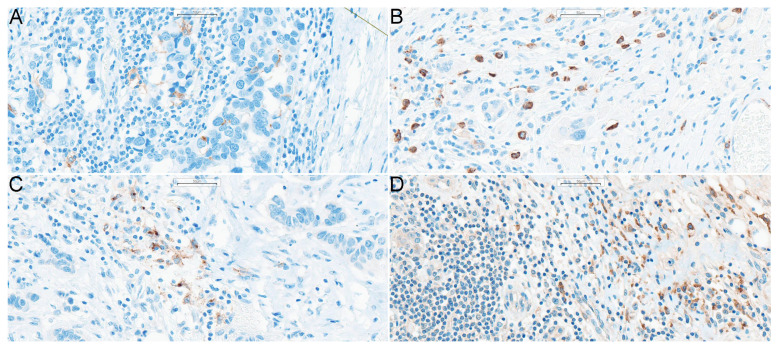
DCs in breast cancer tissue samples. Magnification 200x. Stained DCs shown on particular panels are: (**A**) CD1a^+^ DCs, (**B**) CD123^+^ DCs, (**C**) DC-LAMP^+^ DCs, (**D**) DC-SIGN^+^ DCs. **Abbreviations**: CD1a—cluster of differentiation 1a, CD123—cluster of differentiation 123, DCs—dendritic cells, DC-LAMP—dendritic-cell-lysosome-associated membrane glycoprotein, DC-SIGN—dendritic-cell-specific intercellular-adhesion-molecule-3-grabbing non-integrin, DCIS—ductal carcinoma in situ.

**Figure 2:**
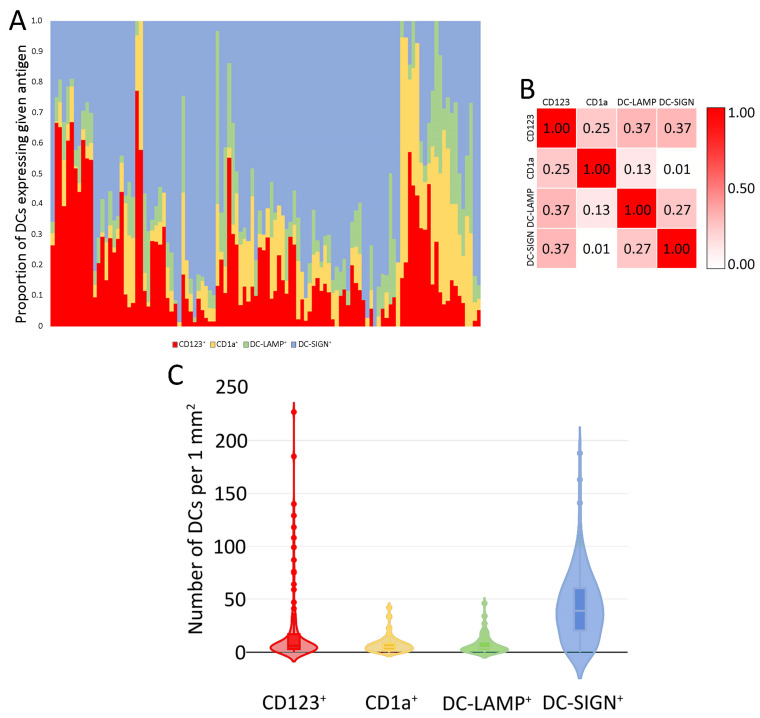
The characteristic of DCs infiltrating in the investigated samples of BC tissues. (**A**) The proportion of DCs with particular superficial antigens in each individual sample. Samples were clustered according to the Euclidean distances with single linkage rule. (**B**) Correlation matrix of the densities of particular DC subpopulations (per 1 mm^2^). Spearman correlation coefficient R was used. Because all correlations were positive, the scale bar ranges from 0 to 1. (**C**) Violin plot of the densities of DC subpopulations. The horizontal lines correspond to the median, boxes represent the interquartile range and the whiskers show the min.-max. range. **Abbreviations**: CD1a—cluster of differentiation 1a, CD123—cluster of differentiation 123, DCs—dendritic cells, DC-LAMP—dendritic-cell-lysosome-associated membrane glycoprotein, DC-SIGN—dendritic-cell-specific intercellular-adhesion-molecule-3-grabbing non-integrin.

The clinicopathological characterization of the study group is summarized in Table 1 (the detailed list of drugs used in NAC is presented in Supplementary Table S1). The representative images showing immunohistochemically stained DCs are presented in Figure 1, while the characteristic of the DCs subpopulations is presented in Figure 2.

**Figure 3:**
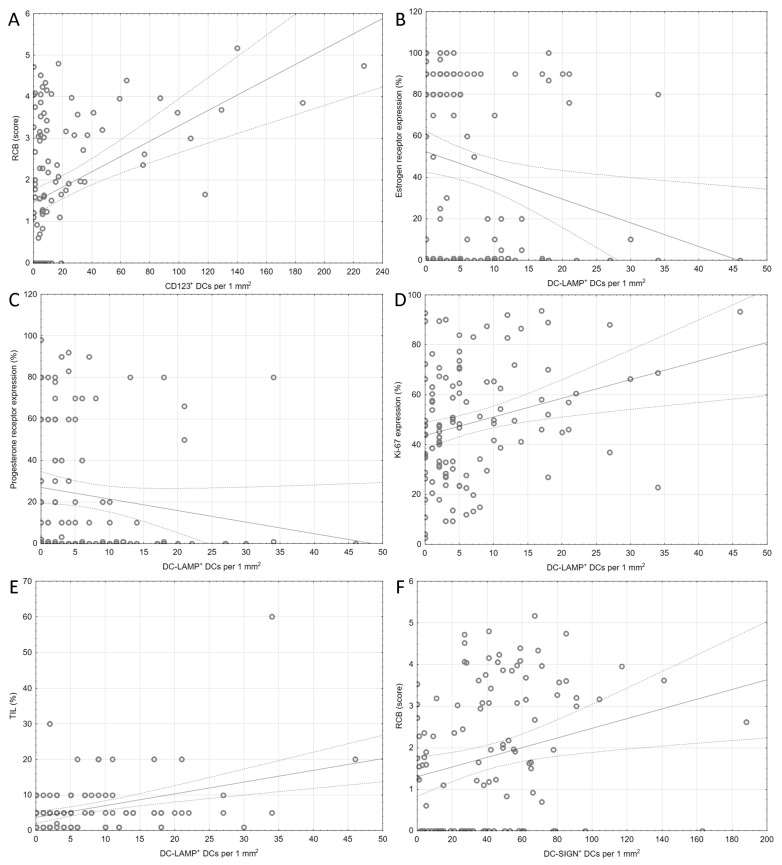
Plots depicting the correlations from Table 2, which remained significant after the Benjamini–Hochberg correction for multiple comparisons. (**A**) CD123^+^ DC density and RCB score, (**B**) DC-LAMP^+^ DC density and estrogen receptor expression, (**C**) DC-LAMP^+^ DC density and progesterone receptor expression, (**D**) DC-LAMP^+^ DC density and Ki-67 expression, (**E**) DC-LAMP^+^ DC density and TILs, (**F**) DC-SIGN^+^ DC density and RCB score. **Abbreviations**: CD1a—cluster of differentiation 1a, CD123—cluster of differentiation 123, DCs—dendritic cells, DC-LAMP—dendritic-cell-lysosome-associated membrane glycoprotein, DC-SIGN—dendritic-cell-specific intercellular-adhesion-molecule-3-grabbing non-integrin, RCB—residual cancer burden, TIL—tumor infiltrating lymphocytes.

In the evaluated samples, the densities of CD123^+^ and DC-SIGN^+^ DCs showed correlation with RCB (R = 0.38, p^BH^ < 0.001 and R = 0.28, p^BH^ = 0.021, respectively). DC-LAMP^+^ DCs were associated with the expression level of Ki-67 assessed in the core-needle biopsy taken before chemotherapy, as well as with TILs density. Simultaneously, they were negatively correlated with the expression of ER and PR. The results of the correlation analysis are shown in Table 2 and Figure 3.

The median density of CD123^+^ DCs was lower in tumors that showed pCR to NAC (p^BH^ = 0.003) and were assessed as RCB class 0 (p^BH^ = 0.001, compared to classes 2 and 3 in post hoc comparisons by the Dunn test). All patients with pCR had ≤ 20 CD123^+^ pDCs per 1 mm^2^. Consequently, a lower number of CD123^+^ DCs was observed in tumors with ypT0 (or in situ disease, p^BH^ = 0.0022), ypN0 (p^BH^ = 0.03), without vascular invasion (p^BH^ = 0.04) and lower grade after chemotherapy (p^BH^ < 0.001). HER2+ tumors had more abundant CD123^+^ DCs (p^BH^ = 0.022).

Differences in the number of DC-LAMP^+^ DCs were associated with the pathological response, the RCB class, and the grading after NAC. However, no clear trends were identified in intergroup comparisons with post-hoc tests.

The results of the intergroup comparisons are presented in Table 3 and Table 4 and Table S2.

In the multivariate stepwise logistic regression, a set of clinical and histopathological features was investigated in order to find the best model capable of predicting pCR. After the univariate analysis, the predictors were chosen and the best model was selected according to the Nagelkerke pseudo-R^2^ value and the degree of fit of the model. The selected predictors included histological and molecular type of tumor, grading and Ki-67 expression before chemotherapy, TIL density, HER2 status, menopausal status, ER and PR expression, number of chemotherapy cycles and the density of CD123^+^ DCs. Other parameters, such as the densities of the remaining subpopulations of the DC, age at the beginning of treatment, and chemotherapy regimen, were not incorporated into the final model.

For the increase in CD123^+^ DCs density by 1 cell per mm^2^ the odds of pCR were reduced by 13% (*p* = 0.006). Additionally the presence of pCR was associated with menopausal status, i.e., menopause reduced odds of pCR by approximately 89% (*p* = 0.006).

The complete description of the logistic regression model is shown in Table 5.

## 3. Discussion

In this observational study, we intended to identify the relationships between the immunohistochemical signatures of various DC subpopulations and the response to NAC in patients with primary BC. We determined that the decrease in CD123^+^ DC density was associated with a more prevalent pCR, lower tumor size and nodal burden and RCB, as well as nuclear grade. Furthermore, HER2-negative tumors showed a higher abundance of CD123^+^ DCs. Ultimately, we proposed a logistic regression model that incorporated remarkable clinical and histological characteristics and found that only menopausal status and CD123^+^ DC density were significant predictors of pCR.

Additionally, we noted some relationships between the density of DC-LAMP^+^ DCs and various histological characteristics; however, the magnitudes of correlations were small and observed dependencies were not consistent across intergroup comparisons.

The density of DC-SIGN^+^ DCs showed only a weak correlation with the RCB score, while we did not identify any association with the density of CD1a^+^ DCs.

Both scientific research and the clinical setting would benefit from tools that allow for a valid prediction of disease-free survival after NAC in BC.

Currently, pCR is most frequently considered as the surrogate outcome, although this approach is still investigated [39,40]. Many studies incorporated advanced systems with genetic profiling or adaptation of convolutional neural networks to predict pCR after NAC [41,42,43]. Nonetheless, biomarkers of BC response to NAC are now scarce and need to be further established.

Decisions regarding the introduction of NAC have to take into account the risk of complications, both typical for common use of chemotherapy, but also specific due to the following surgery. Although some trends towards higher frequency of postoperative wound complications, skin (or nipple) necrosis and formation of seroma were observed, they were not found to be statistically significant [44,45]. Some early reports highlighted the elevated risk of loco-regional recurrence of the disease after NAC, however, their results were biased due do incorporation of data from patients that had only local radiotherapy and no surgery at all [46]. This issue was put back into question as the large meta-analysis of Early Breast Cancer Trialists’ Collaborative Group found a relative risk of 1.37 for the local recurrence of BC (within 15 years from initial treatment) compared to patients who were treated with mastectomy and following adjuvant therapy [47]. Moreover, BCT should only be considered when the tumor response to NAC was the overall shrinkage of its mass, without the islands off scattered (even if singular) tumor cells in the primary tumor site. Currently, repeated magnetic resonance imaging should be considered to address these issues, according to the ESMO guidelines [14].

CD123 is commonly known as the α chain of the interleukin 3 receptor (IL3RA). This superficial antigen is expressed on the part of normal stem cells and is preserved in the granulocytic and monocytic lineage [48]. It belongs to the beta common (β_C_) family of receptors, being responsible for the regulation of growth, proliferation, survival, and differentiation of hematopoietic cells [49]. The activation of the receptor via its classic IL-3 ligand leads to signal transduction with the JAK2/STAT, Ras-MAPK and PI3K pathways [50]. Most current research focused on its role in hematologic malignancies–not only as a biomarker, but also as a powerful target for therapy [51,52]. Of note, CD123 is recognized as the hallmark of the pDCs (and their neoplasms) [53] and is considered as sufficient for their identification [54].

Plasmacytoid DCs share some functionalities with T lymphocytes, i.e., they express CD4 and pre-T cell receptor. They use the toll-like receptor 7 (TLR7) and TLR9 as viral and bacterial nucleic acid sensors, releasing high amounts of type I interferon (INF) upon such stimuli [55]. However, in the steady state, due to the high expression of blood dendritic cell antigen-2 (BDCA-2), pDCs are actually more inclined to be initiators of immune tolerance and can promote T_reg_ differentiation and activate Foxp3^+^ T_reg_ lymphocytes through the indoleamine 2,3-dioxygenase (IDO)-dependent or inducible costimulator-ligand (ICOSL) dependent pathways [56,57]. Both impaired and the prolonged secretion of IFN-α might be responsible for these effects [29,58]. Current research suggests that interaction with other subtypes of DCs is essential for some actions of pDCs, i.e., to cross-prime antigen-specific CD8^+^ T lymphocytes [59].

Multiple studies showed contradictory results regarding the prognostic meaning of pDCs in different malignancies [60,61,62,63,64]. This might be partly explained by the additional heterogeneity observed across pDCs, as they could be further subdivided depending on their expression of CD2, programmed death-ligand 1 (PD-L1) and CD80 [65].

Plasmacytoid DCs were proposed to be attracted to the tumor microenvironment through the CCL20/CCR6 axis [66] or the hypoxia and hypoxia inducible factor-1α (HIF-1α) pathway [67].

Due to the cross-sectional design of this study, it is virtually impossible to determine the exact mechanism resulting in the higher abundance of CD123^+^ pDCs in the tumors that did not response completely to NAC. It could be stipulated that the immunotolerance promoted by pDCs protected tumor cells from the aggravated immune response after chemotherapy. On the contrary, the remaining cancer tissue might have attracted pDCs, which would explain their higher density.

However, all patients with pCR had ≤20 CD123^+^ pDCs per 1 mm^2^ and, for each additional CD123^+^ pDC per 1 mm^2^, the odds of pCR were reduced by approximately 13%. Higher densities of these cells were remarkably associated with higher grade and stage of BC after NAC.

The first insight into the negative prognostic value of pDCs in BC was provided by Treilleux et al., who showed that the infiltrate of CD123^+^ DCs is associated with shorter overall and relapse-free survival. However, the authors used a qualitative scale and considered the CD123^+^ status as simply negative or positive, yet did not provide criteria that would allow replication and easy interpretation of these results. They reported only 13% of the investigated tumors to be infiltrated by pDCs [28]. Data from animal models suggested that pDCs are not mere bystanders and contribute to the variety of immunological processes in the tumor foci [68]. Similarly, a higher density of pDCs coexisted more frequently with BC nodal metastases [69]. The study of the pDC subpopulation in blood samples taken from patients with primary BC revealed higher frequencies of pDCs (as percentage of total blood polymorphonuclears) in those with T0-T1 tumors compared to T2-T4. The authors suggested that this phenomenon was associated with the sequestration of the pDCs in the tumors of the higher stage [70]. We already established that pDCs were more prevalent in high-grade BC approached without any neoadjuvant treatment [37]. Interestingly, our previous findings showed that CD123^+^ pDCs density was particularly increased in higher grade ductal carcinoma in situ (DCIS) showing features of neoduct-genesis [36].

The influence of chemotherapy on the population of tumor-infiltrating pDCs is not well understood. Wagner et al. disclosed that neoadjuvant radio-chemotherapy in rectal cancer resulted in significant increase of pDC frequencies in the tumor stroma, particularly in the population of INF-α^+^ cells [71]. Previous studies investigating the role of pDCs in breast cancer showed that transforming growth factor-β (TGF-β) and tumor necrosis factor-α (TNF-α) abundant in the tumor microenvironment impaired production of INF-α in pDCs [72].

The role of mature DC-LAMP^+^ DCs was rarely investigated in previous research. They were found to be more prevalent in sentinel lymph nodes than in remaining ones, unless nodal macro-metastases were present [73]. Furthermore, one study described their correlation with the smaller tumor size, negative nodal status, positive ER and PR status and, ultimately, with lower nuclear grade. The expression of vascular endothelial growth factor (VEGF) was inversely correlated with the density of mature DCs [74]. In primary invasive BC, we found the highest densities of DC-LAMP^+^ DC in tumors negative for ER and PR [37]. High intra-tumoral DC-LAMP^+^ density was associated with increased odds of multiple lymph node metastases [38]. Additionally, we demonstrated that DC-LAMP^+^ DCs are prevalent in larger tumors with higher nuclear grade and lower expression of ER and PR. Consequently, DCIS with features of neoductgenesis were infiltrated with larger amounts of DC-LAMP^+^ DCs [36]. However, our current findings suggest that the population of DC-LAMP^+^ DCs could not be incorporated as the biomarker of the response to NAC in BC.

Approximately 20–25% of all BC cases have an overexpression of the human epidermal growth factor receptor 2 (HER2), which is associated with more frequent recurrences after initial treatment, as well as higher risk of distant metastases [75].

In all stages of HER2-positive BC molecular subtypes, the basic method of systemic treatment is use of HER2-targeting monoclonal antibodies, such as trastuzumab and pertuzumab, in combination with chemotherapy [76,77]. In addition to their primary role of blocking HER2-driven oncogenic signaling pathways, both antibodies have the ability to stimulate an antitumor immune response [78]. Trastuzumab and pertuzumab modulate the functioning of the immune system because they activate natural killer (NK) cells via the FCγRIII receptor [79]. Furthermore, they potentialize cytotoxic T lymphocytes as they enhance the presentation of HER2 molecule fragments with class I major histocompatibility complex [80,81]. The immunomodulatory effect of trastuzumab is also indicated by activation anti-HER2 CD8^+^ T cell immune response with improved progression-free survival in patients with HER2-positive metastatic BC [82]. Trastuzumab was also observed to induce CD4^+^ helper T cell-associated antitumor immunity in patients with early HER2-positive BC [83].

Polychemotherapy with trastuzumab and pertuzumab in patients with HER2-positive BC is often associated with significant toxicity, and there is increasing interest in de-escalation strategies using techniques that induce a positive immune response in the tumor microenvironment in combination with HER2-targeted antibodies [84].

A special role is assigned to DCs, which are an effective tool for generating an immune response specific to the tumor antigen [85]. An experimental vaccine with pulsed HER2 peptides and DCs polarized against HER2 (HER2-DC1) was capable of potentializing the TH1-dependent immune response against this antigen in subjects with both HER2-positive DCIS and HER2-positive early invasive BC, and improved pCR [86,87].

Administration of DCs immunized against HER2 combined with anti-HER2 antibodies tampers with tumor growth and reduces mortality in the mouse model of HER2-positive BC [88].

In the HER2-positive BC, intra-tumoral delivery of HER2-DC1 complex combined with anti-HER2 antibodies effectively diminished activation of HER2-mediated oncogenic signaling pathways. Studies in mice have shown that intra-tumoral HER2-DC1 enhances the effectiveness of anti-HER2 immunoglobulins more than conventional chemotherapy [89,90]. In HER2-positive BC, it can induce complete tumor regression in 75–80% of treated mice, and the resulting lasting immunity prevented secondary tumor formation [91]. Clinical trials in this area are ongoing.

A remarkable consideration is that BC is a heterogeneous entity with a multitude of molecular, histological, and clinical variants. Although our cumulative analysis made a reasonable attempt to adjust for this variability (i.e., through multiple logistic regression), it has to be emphasized that the magnitude of the observed relationships between response to NAC and DC subpopulations might be different in distinct subgroups, particularly these of high clinical stage or unfavorable molecular subtype. Any strict conclusions should be preceded by further evaluation of the prognostic significance of DCs in these exact populations, with longitudinal studies with larger groups of patients.

## 4. Materials and Methods

### 4.1. Patients

The immunostaining was acquired from BC tissue of BC from 114 women, all of whom had their histological diagnosis between 2015 and 2021 in the Department of Pathomorphology of the University Hospital in Krakow (Poland).

First, all patients had core-needle biopsy from which the diagnosis of BC was established by an experienced pathologist. After the diagnosis, all patients underwent NAC, a regimen selected based on the ESMO guidelines [14] in the Oncology Clinical Department of University Hospital in Krakow. After NAC, the patients were qualified and operated upon in the Breast Unit of the University Hospital in Krakow. The surgical material was then assessed in the Department of Pathomorphology.

Inclusion criteria for the study were: (1) female gender, (2) being diagnosed with BC, treated with neoadjuvant chemotherapy (without hormonotherapy) and qualified for surgical treatment afterwards, (3) no distant metastases of BC and (4) no other malignancy at the initial diagnosis of breast cancer. Patients that fulfilled the above criteria were identified in the registry of University Hospital and qualified for the study. Basic patient demographics, surgical data, regimen and duration of NAC and routine histological data were received from hospital records (Table 1). The clinical stage (c) before NAC, the pathological stage after it (yp) and the pathological response were established according to the 8th edition of AJCC guidelines from 2017 [92]. The Nottingham Histologic Grade system was used for grading. Residual Cancer Burden Calculator was used to determine RCB score and class [93].

Routinely processed formalin-fixed, paraffin-embedded tissue was retrieved from the archive and processed as described in the Immunohistochemical techniques section.

The study was conducted with respect to the principles set out in the Helsinki Declaration of 1964, as revised in 1983. The study protocol was approved by the Bioethics Committee of Jagiellonian University (1072.6120.289.2020 from 28 October 2020).

### 4.2. Immunohistochemical Techniques

Immunohistochemistry for CD1a, CD123, DC-LAMP, DC-SIGN, ER, PR, HER2 and Ki-67 was performed according to the protocol routinely used in our laboratory (Table 6). Immunostaining for CD1a, DC-LAMP, DC-SIGN, ER, PR and HER2/neu was performed automatically on BenchMark Ultra immunostainer (Roche Ventana, Tucson, AZ, USA) and immunostaining for CD123 and Ki-67 was performed automatically on DAKO Omnis immunostainer (Dako, Santa Clara, CA, USA). The control tissues for immunohistochemistry were tonsils for CD123, DC-LAMP and DC-SIGN, breast for ER and PR and breast cancer for HER2 (both negative and positive control). For Ki-67 tonsils, appendix, pancreas and liver were control tissues. For CD1a, skin was positive and thyroid gland, placenta, and prostate were negative control tissues.

Positive expression of ER and PR was set at ≥1% of tumor cells showing positive nuclear immunostaining. The threshold to discriminate between low and high Ki-67 expression was set at ≥20% of positive cells. Scoring of the HER2 staining was performed by standard method [94].

For specimens with HER2 status 2+ in immunohistochemistry, fluorescence in situ hybridization (FISH) was conducted. FISH was performed using a ZytoLight FISH-Tissue Implementation Kit (ZYTOVISION GmbH, Bremerhaven, Germany) according to the manufacturer’s protocol. The CytoHYB CT500 automatic system (CytoTest Inc., Rockville, MD, USA) was used for denaturation and hybridization. The Locus Specific Identifier HER-2/neu and CEP17 signals were counted using a fluorescence microscope equipped with specific filter sets. HER2/neu overexpression was identified when the signal ratio of HER-2/neu to CEP17 was >2.0 [94].

### 4.3. Histologic DC Scoring and Analysis

First, virtual slides were acquired on the Aperio GT 450 DX scanner (Leica Biosystems, USA) for each immunostained slide. The virtual slides were then visualized and analyzed in the MedLan Slide Viewer software v.1.11 (MedLan, Poland). In each virtual slide, the areas with the highest number of cells positive for CD1a, CD123, DC-SIGN, and DC-LAMP (“hotspots”) were chosen at low magnification (100–250×). Using the software tools, hotspot areas with a total of approximately 5 mm^2^ were framed, and positively immunostained cells were labeled and counted within selected hot-spot areas. For RCB 0 the DCs were counted in the area of post-tumoral cicatrix while for RCB 1–3 classes they were counted in peritumoral tumor stroma of approximately 5 mm^2^ area (measured exactly for each case and staining). DCs located in close proximity to the epidermis or dermis were excluded. The final result was the sum of positively-stained cells obtained in each slide from each selected area calculated per 1 mm^2^ of tumor tissue.

To avoid misinterpretation of nonspecifically stained elements as DCs, only cells with strong cytoplasmatic staining, visible nuclei, and characteristic morphological features, such as irregular dendritic appearance of cells for CD1a^+^, DC-LAMP3^+^ and DC-SIGN^+^ DCs, were counted, as well as round-shaped cells without protrusions for CD123^+^ DCs.

### 4.4. Statistical Elaboration

Nominal data were shown as absolute and relative frequencies (N, %), whereas quantitative data were described with median and standard deviation, or the min.–max. range. Distribution of interval data was assessed through the visual assessment of histograms and with Shapiro–Wilk’s test for normality. Because, for any intergroup comparison, the distribution of the data within groups was skewed, only nonparametric tests were used for further analyses. The relationship between quantitative data was described with the Spearman correlation coefficient R. Differences between two groups were assessed with the *U* Mann-Whitney’s test and, if there were more than two groups, the Kruskal–Wallis ANOVA with post hoc Dunn’s test was used. Multivariate stepwise logistic regression was used to find the predictors of pCR to NAC. The optimal model was selected with respect to the highest Nagelkerke pseudo-R^2^ and the best fit according to the Hosmer–Lemeshow test.

As a threshold for significance, α was decided to be 0.05 in all analyses. To avoid false positive results caused by multiple comparisons, a Benjamini–Hochberg correction was used with the assumption of a false discovery ratio 0.05, and corrected *p*-values (p^BH^) were also reported.

All statistical analyses were conducted in Statistica 13.3 software (Statsoft Inc., Tulsa, OK, USA).

## 5. Conclusions

Analysis of DC subpopulations in the postoperative material from BC surgery after NAC confirmed that pDCs, identified by the presence of CD123^+^ antigen, are a substantial component of the tumor microenvironment. Their abundance is related to numerous indicators of a worse prognosis and progression of the disease. Ultimately, the density of CD123^+^ pDCs showed its capability to predict lack of pCR in the studied group. We identified a few relationships with the population of DC-LAMP^+^ DCs, although their potential significance remains unclear.

Our findings open new opportunities for future researchers. Additional evaluation of the DC subpopulations before NAC would allow investigation of the meaning of absolute and relative changes in their composition.

Finally, although pCR is widely considered a surrogate endpoint for survival in patients with BC, it is not a perfect measure [95]. Therefore, prospective trials are needed that would assess survival and relapse-free time with respect to the density measured of CD123^+^ pDCs to provide definite evidence. Ultimately, NAC is associated with numerous short- and long-term complications—the biomarkers of response to it may also be useful in predictions of such adverse reactions, thus supporting a clinical decision-making process.

## Figures and Tables

**Table 1:** Baseline characteristics of the study group. Interval data are shown as median (min.-max.) range or as mean ± standard deviation, while nominal data are presented as absolute and relative frequencies: N (%).

Characteristic		Value
**Clinical Data**		
Age at diagnosis (years)		51.5 (28–85)
Menopausal status	premenopausal	54 (49%)
	postmenopausal	57 (51%)
Time from diagnosis to surgical treatment (months)		6 (3–9)
*Surgery type*		
BCT		60 (53%)
Mastectomy		54 (47%)
*Lymph nodes surgery*		
SLNB		64 (56%)
ALND		50 (44%)
*cT stage*		
cT1		9 (8%)
cT2		61 (54%)
cT3		28 (25%)
cT4		15 (13%)
*cN stage*		
cN0		40 (35%)
cN1		61 (54%)
cN2		4 (4%)
cN3		8 (7%)
NAC cycles (number)		8 (4–18)
*Chemotherapeutics used in NAC*		
Anthracyclines		105 (92%)
Taxanes		107 (94%)
Platinum (IV) derivatives		33 (29%)
Cyclophosphamide		101 (87%)
*Immune therapy*		
Trastuzumab		21 (18%)
Trastuzumab + pertuzumab		17 (15%)
**Histological data**		
TIL (%)		5 (1–60)
*Histological type*		
NST		95 (83%)
ILC		6 (5%)
other		13 (12%)
*Molecular subtype*		
luminal A		4 (4%)
luminal B HER2-		42 (37%)
luminal B HER2+		26 (23%)
non-luminal HER2+		15 (13%)
TNBC		27 (24%)
Ki-67 (percent in core-needle biopsy)		49.0 ± 23.0
RCB score		1.7 (0–5.2)
*RCB class*		
0		40 (35%)
I		11 (10%)
II		37 (32%)
III		26 (23%)
*Pathological response*		
pCR		40 (35%)
pPR		62 (54%)
pNR		12 (11%)
*Nuclear grade (before NAC)*		
G1		7 (6%)
G2		51 (45%)
G3		56 (49%)
*Nuclear grade (after NAC)*		
G0		40 (39%)
G1		9 (9%)
G2		39 (39%)
G3		13 (13%)
*HER2 status*		
(+)		42 (37%)
(−)		71 (63%)
ER (%)		50 (0–100)
PR (%)		1 (0–98)
*ypT stage*		
ypT0		34 (30%)
DCIS		8 (7%)
ypT 1		43 (37%)
ypT 2		22 (19%)
ypT 3		4 (4%)
ypT 4		3 (2%)
*ypN stage*		
ypN0		70 (61%)
ypN1mi		2 (2%)
ypN1		19 (17%)
ypN2		16 (14%)
ypN3		7 (6%)
Vascular invasion (post-NAC), n (%)		45 (39%)

**Abbreviations:** ALND—axillary lymph nodes dissection, BCT—breast-conserving therapy, DCIS—ductal carcinoma in situ, ER—estrogen receptor, HER2—human epidermal growth factor receptor 2, ILC—invasive lobular carcinoma, NAC—neoadjuvant chemotherapy, NST—no special type, PR—progesterone receptor, pCR—pathological complete response, pNR—pathological no response, pPR—pathological partial response, RCB—residual cancer burden, SLNB—sentinel lymph node biopsy, TIL—tumor infiltrating lymphocytes, TNBC—triple-negative breast cancer.

**Table 2:** Correlation analysis of the relationships with the density of the superficial dendritic cell markers (per 1 mm^2^). The Spearman correlation coefficient R (above) is given together with the p/p^BH^ values (below).

Characteristic	CD123^+^	CD1a^+^	DC-LAMP^+^	DC-SIGN^+^
Age (years)	−0.07 0.4/-	−0.22 0.015/0.09	0.02 0.9/-	0.03 0.8/-
Number of chemotherapy cycles	−0.14 0.1/-	−0.04 0.7/-	0.08 0.4/-	−0.09 0.3/-
TIL (%)	0.17 0.08/-	−0.08 0.4/-	0.28 0.003/0.021	0.14 0.1/-
Ki-67 before chemotherapy	−0.02 0.9/-	0.23 0.016/0.09	0.24 0.01/0.04	−0.03 0.8/-
Estrogen receptor expression	0.14 0.1/-	−0.08 0.4/-	−0.24 0.01/0.04	0.02 0.8/-
Progesterone receptor expression	0.07 0.5/-	0.00 1.0/-	−0.22 0.02/0.04	0.00 1.0/-
RCB	0.38 <0.001/<0.001	−0.03 0.8/-	−0.10 0.3/-	0.28 0.003/0.021

**Abbreviations:** CD1a—cluster of differentiation 1a, CD123—cluster of differentiation 123, DC-LAMP—dendritic-cell-lysosome-associated membrane glycoprotein, DC-SIGN—dendritic-cell-specific intercellular-adhesion-molecule-3-grabbing non-integrin, p/p^BH^—*p*-value and *p*-value after Benjamini–Hochberg correction (respectively), RCB—residual cancer burden, TIL—tumor infiltrating lymphocytes.

**Table 3:** Comparison between dichotomous characteristics and superficial dendritic cell markers’ density (per 1 mm^2^). All comparisons were performed with U Mann–Whitney test due to the non-normal distribution of the data. Values are shown as median (min.-max. range) with the p/p^BH^ values.

Characteristic		CD123^+^	p/p^BH^-Value	CD1a^+^	p/p^BH^-Value	DC-LAMP^+^	p/p^BH^-Value	DC-SIGN^+^	p/p^BH^-Value
HER2-negative HER2-positive		8 (0–227) 5 (0–140)	0.004/0.022	6 (0–42) 5 (0–33)	0.3/-	4 (0–34) 4 (0–46)	0.8/-	41 (0–188) 33 (0–163)	0.3/-
ypT0-is ypT1–4		5 (0–19) 9 (0–227)	0.0002/0.0022	5 (0–33) 5.5 (0–42)	0.6/-	5.5 (0–46) 3 (0–34)	0.003/0.03	28 (0–163) 43.5 (0–188)	0.04/-
ypN0 ypN1–3		5 (0–118) 9 (0–227)	0.008/0.03	5 (0–34) 5 (0–42)	0.8/-	4.5 (0–46) 3 (0–34)	1.0/-	29.5 (0–188) 48 (0–141)	0.009/0.1
vascular invasion	no yes	5 (0–227) 9 (0–185)	0.014/0.04	6 (0–42) 5 (0–23)	0.5/-	5 (0–46) 3 (0–34)	0.2/-	29.5 (0–188) 46 (0–141)	0.08/-

**Abbreviations:** CD1a—cluster of differentiation 1a, CD123—cluster of differentiation 123, DC-LAMP—dendritic-cell-lysosome-associated membrane glycoprotein, DC-SIGN—dendritic-cell-specific intercellular-adhesion-molecule-3-grabbing non-integrin, HER2—human epidermal growth factor receptor 2, p/p^BH^—*p*-value and *p*-value after Benjamini–Hochberg correction (respectively).

**Table 4:** Comparison between non-dichotomous characteristics and superficial dendritic cell markers (per 1 mm^2^). All the comparison were performed with Kruskal–Wallis test due to the non-normal distribution of the data. Values are shown as median (range) with the p/p^BH^ values.

Characteristic		CD123^+^	p/p^BH^-Value	CD1a^+^	p/p^BH^-Value	DC-LAMP^+^	p/p^BH^-Value	DC-SIGN^+^	p/p^BH^-Value
Histological type	NST lobular others	6 (0–185) 5.5 (0–17) 16 (1–227)	0.06/-	5 (0–42) 5 (1–10) 8 (0–16)	0.3/-	4 (0–46) 1.5 (0–8) 3 (0–27)	0.2/-	38 (0–141) 34 (0–80) 49 (0–188)	0.7/-
Molecular subtype	luminal A luminal B HER2- luminal B HER2+ non-luminal HER2+ TNBC	3 (0–6) 10 (0–185) 5 (0–140) * 3 (0–87) * 6 (0–227)	0.024/0.03	1.5 (0–5) 7 (0–23) 4.5 (0–16) 5 (0–33) 5.5 (0–42)	0.2/-	2 (0–7) 2.5 (0–34) 4 (0–18) 4 (0–46) 5 (0–34)	0.2/-	46.5 (28–59) 41.5 (0–188) 32.5 (0–163) 32 (4–91) 29 (1–117)	0.7/-
anty-HER2 treatment	no treatment trastuzumab trastuzumab + pertuzumab	9 (0–227) *^,#^ 3 (0–22) * 5 (0–47) ^#^	0.0001/0.0005	5.5 (0–42) 5 (0–16) 5 (0–33)	1.0/-	5 (0–34) 2 (0–10) 6 (0–46)	0.04/0.08	42 (0–188) 15 (0–71) 29 (6–163)	0.008/0.08
grading after chemotherapy	0 1 2 3	5 (0–19) * 15 (0–26) 6 (0–227) ^#^ 59 (6–185) *^,#^	<0.0001/<0.001	5 (0–33) 5 (0–18) 5 (0–42) 7 (1–23)	0.8/-	5 (0–46) * 2 (0–8) ^#^ 2 (0–34) *^,†^ 11 (2–22) ^#,†^	0.0004/0.002	29 (2–163) 28 (0–104) 41 (0–141) 57 (0–188)	0.039/0.1
cT	1 2 3 4	3 (0–108) 7 (0–227) 6 (0–118) 12 (0–140)	0.2/-	6 (0–42) 5 (0–33) 7 (0–14) 5 (0–34)	0.9/-	4 (1–34) 4 (0–46) 3 (0–21) 5 (0–30)	0.3/-	39 (0–91) 37.5 (0–163) 34 (0–141) 49 (1–188)	0.5/-
cN	0 1 2 3	4 (0–118) * 8 (0–227) * 0.5 (0–59) 5 (1–140)	0.0174/0.023	5 (0–34) 6 (0–42) 2 (1–3) 3.5 (0–15)	0.3/-	4 (0–46) 4 (0–34) 2 (0–22) 12.5 (1–30)	0.2/-	27 (0–188) 41 (0–163) 69.5 (34–117) 51 (13–81)	0.07/-
ypT	0 is 1 2 3 4	5 (0–12) * 3.5 (0–19) 7 (0–118) 15.5 (0–227) * 12.5 (5–76) 6 (5–140)	0.011/0.018	5 (0–33) 7.5 (2–23) 6 (0–42) 4 (0–23) 10.5 (3–11) 8 (0–14)	0.4/-	5 (0–46) 5.5 (2–17) 2 (0–34) 4.5 (0–34) 3 (0–11) 7 (3–14)	0.012/0.03	27.5 (2–96) 34 (0–163) 41 (0–141) 45.5 (0–117) 55 (27–188) 49 (47–67)	0.2/-
ypN	0 mi 1 2 3	5 (0–118) * 17 (6–28) 34 (1–227) * 6.5 (0–87) 5 (0–140)	0.0023/0.005	5 (0–34) 15.5 (8–23) 7 (0–42) 3.5 (0–14) 5 (1–15)	0.1/-	4.5 (0–46) 14.5 (12–17) 4 (0–34) 2.5 (0–18) 1 (0–14)	0.2/-	29.5 (0–188) 54 (51–57) 41 (0–141) 53 (11–85) 41 (0–80)	0.8/-
pathological response	pCR pPR pNR	5 (0–19) *^,#^ 9 (0–227) * 8.5 (0–140) ^#^	0.0011/0.003	5 (0–33) 6 (0–42) 5 (0–11)	0.4/-	5.5 (0–46) * 2.5 (0–34) * 7.5 (0–34)	0.007/0.02	28 (2–163) 41.5 (0–188) 53 (0–141)	0.1/-
RCB class	0 1 2 3	5 (0–19) *^,#^ 6 (0–18) 10 (0–118) * 10.5 (0–227) ^#^	0.0003/0.001	5 (0–33) 6 (1–23) 7 (0–42) 4 (0–23)	0.2/-	5.5 (0–46) *^,#^ 1 (0–17) *^,†^ 2 (0–34) ^#^ 6.5 (0–34) ^†^	0.0003/0.003	28 (2–163) 38 (0–71) 41 (0–188) 51.5 (0–141)	0.028/0.1

Symbols (*,^#^,^†^) were used to indicate intergroup differences established through the Dunn post hoc test in the comparisons that remained statistically significant after Benjamini-Hochberg correction for false discoveries. **Abbreviations:** CD1a—cluster of differentiation 1a, CD123—cluster of differentiation 123, DC-LAMP—dendritic-cell-lysosome-associated membrane glycoprotein, DC-SIGN—dendritic-cell-specific intercellular-adhesion-molecule-3-grabbing non-integrin, HER2—human epidermal growth factor receptor 2, NST– no special type, pCR—pathologic complete response, pPR—pathologic partial response, pNR—pathologic no response, p/p^BH^—*p*-value and *p*-value after Benjamini–Hochberg correction (respectively), RCB—residual cancer burden, TNBC–triple-negative breast cancer.

**Table 5:** Logistic regression model predicting pCR with respect to the evaluated predictors.

Characteristic		OR (95%CI)	*p*-Value
Histological type	NST other	2.08 (0.34–12.65) reference	0.4
Molecular subtype	TNBC other	9.45 (0.95–94.27) reference	0.056
Grading before chemotherapy		2.99 (0.94–9.49)	0.063
Ki-67 before chemotherapy		1.01 (0.98–1.04)	0.5
TIL (%)		1.11 (0.96–1.30)	0.2
HER2 status	(+) (−)	3.30 (0.67–16.21) reference	0.1
menopausal status	(+) (−)	0.11 (0.02–0.53) reference	0.006
Estrogen receptor expression		0.99 (0.97–1.02)	0.5
Progesterone receptor expression		0.98 (0.95–1.02)	0.4
Number of chemotherapy cycles		0.91 (0.75–1.10)	0.3
CD123^+^ cells/mm^2^		0.87 (0.79–0.96)	0.006

OR for all continuous and ordinal variables is given per 1 unit of change. Nagelkerke pseudo-R^2^ = 0.61; Hosmer–Lemeshow test *p*-Value > 0.9. **Abbreviations:** CD123—cluster of differentiation 123; CI—confidence interval; HER2—human epidermal growth factor receptor 2; NST—no special type; OR—odds ratio; TIL—tumor infiltrating lymphocytes; TNBC—triple-negative breast cancer.

**Table 6:** Characteristics of antibodies used in the study.

Antibody	Clone	Dilution	Antigen Retrieval	Incubation Time	Manufacturer	Detection System
CD1a	polyclonal	8:100	Citrate	36 min	Novocastra	ultraView Universal DAB Detection Kit (Roche Ventana)
CD123	polyclonal	1:100	EDTA	60 min	Novocastra	OptiView DAB IHC Detection Kit (Roche Ventana)
DC-LAMP	polyclonal	2:100	ULTRA CC1 (Roche Ventana)	72 min	NovusBio	ultraView Universal DAB Detection Kit (Roche Ventana)
DC-SIGN	5D7	2:100	ULTRA CC1 (Roche Ventana)	72 min	Abcam	ultraView Universal DAB Detection Kit (Roche Ventana)
ER	SP1	RTU	EDTA	16 min	Roche	ultraView Universal DAB Detection Kit (Roche Ventana)
PR	1E2	RTU	EDTA	16 min	Roche	ultraView Universal DAB Detection Kit (Roche Ventana)
Ki67	MIB-1	RTU	EDTA	20 min	Dako, USA	ultraView Universal DAB Detection Kit (Roche Ventana)
HER2/neu	4B5	RTU	Citrate	16 min	Roche	ultraView Universal DAB Detection Kit (Roche Ventana)

## Data Availability

The data presented in this study are available on request from the corresponding author. The data are not publicly available due to privacy restrictions.

## Supplementary Materials

The supporting information can be downloaded at: https://www.mdpi.com/article/10.3390/ijms242115817/s1.

## Abbreviations

APCs	antigen presenting cells
BC	breast cancer
BDCA	blood dendritic cell antigens
cDCs	conventional dendritic cells
CMF	cyclophosphamide/methotrexate/5-fluorouracil
DCIS	ductal carcinoma in situ
DCs	dendritic cells
DC-LAMP	dendritic-cell-lysosome-associated membrane glycoprotein
DC-SIGN	dendritic-cell-specific intercellular-adhesion-molecule-3-grabbing non-integrin
ER	estrogen receptor
FISH	fluorescence in situ hybridization
Flt3L	FMS-like tyrosine kinase 3 ligand
HER2	human epidermal growth factor receptor 2
HIF-1α	hypoxia inducible factor-1α
ICOSL	inducible costimulatory ligand
IDO	indoleamine 2,3-dioxygenaze
IFN	interferon
MHC	major histocompatibility complex
NAC	neoadjuvant chemotherapy
NK cells	natural killer cells
pCR	pathological complete response
pDCs	plasmacytoid dendritic cells
PD-L1	programmed death-ligand 1
PR	progesterone receptor
PRRs	pattern recognition receptors
RCB	residual cancer burden
SLNs	sentinel lymph nodes
TGF-β	transforming growth factor-β
TLR	toll-like receptor
TNBC	triple-negative breast cancer
TNF-α	tumor necrosis factor-α
VEGF	vascular endothelial growth factor
