# Supplementary material for: Dendritic Cell Subpopulations Are Associated with Prognostic Characteristics of Breast Cancer after Neoadjuvant Chemotherapy—An Observational Study
Source: Int J Mol Sci. 2023 Oct 31;24(21):15817. doi: 10.3390/ijms242115817 (PMC10648319; doi:10.3390/ijms242115817)
Supplement: Supplementary file 1 [file ijms-24-15817-s001.zip › ijms-2671371-supplementary.pdf]

Table S1. Detailed list of the drugs used for neoadjuvant chemotherapy. Data are presented as absolute and relative frequencies: N (%).

Drug	N (%)
Paclitaxel	45 (40%)
Docetaxel	66 (58%)
Carboplatin	29 (25%)
Doxorubicin	101 (89%)
Epirubicin	4 (4%)
Cyclophosphamide	101 (89%)
Trastuzumab	38 (33%)
Pertuzumab	17 (15%)

Table S2. Comparison between non-dichotomous characteristics and superficial dendritic cell markers (per 1 mm²). All the comparison were performed with Kruskal-Wallis test due to the nonnormal distribution of the data. Values are shown as median [range] with the p/p^{BH} values.

Characteristic		CD123^{+}	p/p^{BH}-Value	CD1a^{+}	p/p^{BH}-Value	DC-LAMP^{+}	p/p^{BH}-Value	DC-SIGN^{+}	p/p^{BH}-Value
cT1-2		6 [0-227]	0.4/-	5 [0-42]	0.5/-	4 [0-46]	0.5/-	37.5 [0-163]	0.8/-
cT3-4		6.5 [0-140]		6 [0-34]		4 [0-30]		41 [0-188]	
cN0		5 [0-118]	0.032/0.07	4.5 [0-34]	0.9/-	4 [0-46]	0.4/-	29 [0-188]	0.08/-
cN1-3		7 [0-227]		5 [0-42]		5 [0-34]		42 [0-163]	
premenopausal		7 [0-140]	0.6/-	6 [0-33]	0.3/-	4.5 [0-46]	0.9/-	36 [0-163]	0.5/-
postmenopausal		6 [0-227]		5 [0-42]		4 [0-34]		42 [0-188]	
anthracyclines	no	7 [1-99]	0.5/-	3 [0-11]	0.5/-	1 [0-20]	0.1/-	36 [9-188]	0.7/-
	yes	6 [0-227]		5.5 [0-42]		4 [0-46]		39 [0-163]	
taxanes	no	17 [0-129]	0.4/-	3 [0-9]	0.2/-	7 [0-34]	0.1/-	59 [28-91]	0.1/-
	yes	6 [0-227]		5.5 [0-42]		4 [0-46]		37 [0-188]	
platinum derivatives	no	6 [0-185]	1.0/-	5 [0-33]	0.5/-	3 [0-46]	0.08/-	41 [0-163]	0.2/-
	yes	6 [1-227]		6 [0-42]		5 [0-34]		27 [1-188]	
cyclophosphamide	no	9 [1-140]	0.3/-	4 [0-11]	0.2/-	2 [0-17]	0.5/-	42 [9-188]	0.09/-
	yes	6 [0-227]		6 [0-42]		4 [0-46]		37 [0-163]	
HER2-negative		8 [0-227]	**0.004/0.022**	6 [0-42]	0.3/-	4 [0-34]	0.8/-	41 [0-188]	0.3/-
HER2-positive		5 [0-140]		5 [0-33]		4 [0-46]		33 [0-163]	
ypT0-is		5 [0-19]	**0.0002/0.0022**	5 [0-33]	0.6/-	5.5 [0-46]	**0.003/0.03**	28 [0-163]	0.04/-
ypT1-4		9 [0-227]		5.5 [0-42]		3 [0-34]		43.5 [0-188]	
ypN0		5 [0-118]	**0.008/0.03**	5 [0-34]	0.8/-	4.5 [0-46]	1.0/-	29.5 [0-188]	0.009/0.1
ypN1-3		9 [0-227]		5 [0-42]		3 [0-34]		48 [0-141]	
vascular invasion	no	5 [0-227]	**0.014/0.04**	6 [0-42]	0.5/-	5 [0-46]	0.2/-	29.5 [0-188]	0.08/-
	yes	9 [0-185]		5 [0-23]		3 [0-34]		46 [0-141]	

Abbreviations: CD1a—cluster of differentiation 1a, CD123—cluster of differentiation 123, DC-LAMP—dendritic-cell-lysosome-associated membrane glycoprotein, DC-SIGN—dendritic-cell-specific intercellular-adhesion-molecule-3-grabbing non-integrin, HER2—human epidermal growth factor receptor 2, p/pBH—p-value and p-value after Benjamini–Hochberg correction (respectively).
